# Supplementary material for: Supramolecular aptamer nano-constructs for receptor-mediated targeting and light-triggered release of chemotherapeutics into cancer cells
Source: Nat Commun. 2018 Feb 7;9:535. doi: 10.1038/s41467-018-02929-2 (PMC5803212; doi:10.1038/s41467-018-02929-2)
Supplement: Supplementary file 1 — Supplementary Information [file 41467_2018_2929_MOESM1_ESM.pdf]

Supplementary Information

Supramolecular aptamer nano-constructs for receptor-mediated targeting and light-triggered release of chemotherapeutics into cancer cells

Deepak K. Prusty[1,2], Volker Adam[1], Reza M. Zadegan[3], Stephan Irsen[4] and Michael Famulok[1,2,*]

[1]Life and Medical Sciences (LIMES) Institute, Chemical Biology & Medicinal Chemistry Unit, c/o Kekulé Institute of Organic Chemistry and Biochemistry, Gerhard-Domagk-Strasse 1, 53121 Bonn, Germany

[2]Stiftung Caesar, Max-Planck-Fellowship group Chemical Biology, Ludwig-Erhard-Allee 2, 53175 Bonn, Germany

[3]Nanoscale Materials & Device group, Micron School of Materials Science and Engineering, Boise State University, USA

[4]Stiftung Caesar, Elektronenmikroskopie und Analytik, Ludwig-Erhard-Allee 2, 53175 Bonn, Germany

Supplementary Methods

Synthesis of lipid-modified 5´-DMTr-2´-deoxyuridine-phosphoramidite

5 -(1-Dodecynyl)-modified 5´-DMT-2´-deoxyuridine-phosphoramidite **1** (Supplementary Fig. 1) was synthesized as reported in a previous study.[1] Prior to the reaction, 5-[dodecynyl]-5′-O-dimethoxytrityl-2′-deoxyuridine (0.2 g, 288 µmol) was dried overnight under vacuum, then dissolved in 7 mL of extra-dry dichloromethane (treated with molecular sieves) under argon atmosphere and the solution was cooled to 0 °C in an ice-bath. Diisopropylethylamine (0.041 g, 316 µmol) was added under Ar atmosphere at 0 °C and the reaction mixture was stirred for 15 min at 0 °C. Then 2-cyanoethyl-*N,N*-diisopropyl-chlorophosphoramidite (0.075 g, 0.51 µmol, 77 µL) was added dropwise at 0 °C under argon atmosphere. The ice-bath was removed after 10 min and the reaction mixture was stirred for 2 h at r.t. under Ar atmosphere. Reaction progress was monitored over time by TLC using 1:1 v/v mixture of Hexane: Ethyl acetate. After completion of the reaction, all solvents were removed in *vacuo* at a reduced temperature of 30 °C. The product was isolated using flash chromatography (silica gel, eluent: cyclohexane/ethyl acetate = 50/50) to give 180 mg (70%) of **1** as a pale yellow foamy solid. The product was confirmed by ESI mass spectrometry and ^{31}P-NMR.

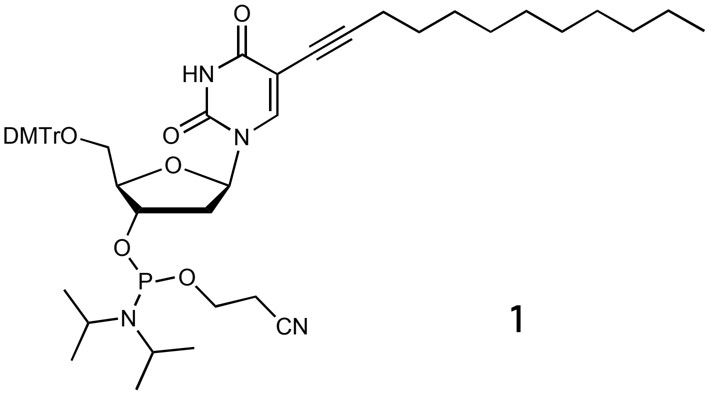

1

Supplementary Figure 1. 5-(1-Dodecynyl) modified 5´-DMTr-2´-deoxyuridine-phosphoramidite **1**

Characterization data: ^{31}P NMR (162 MHz, CD_2Cl_2): δ 149.19 (s), 149.33 (s); HRMS (*m/z*): $[M+H]^+$ calcd. for $C_{51}H_{67}N_4O_8PH$, 895.4769; found, 895.4773

Characterization by ^{31}P NMR

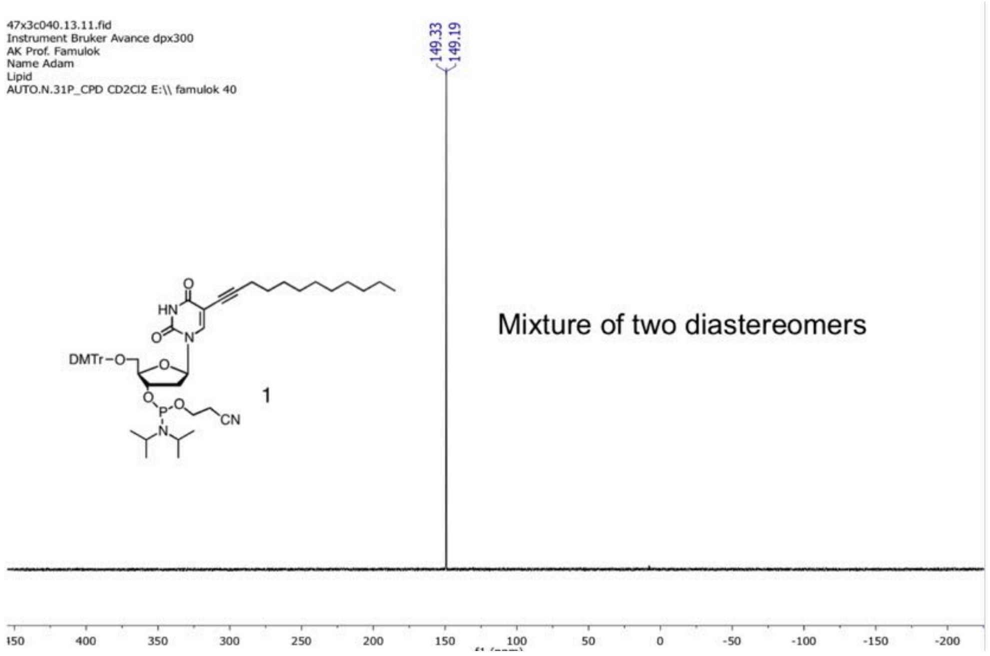

Supplementary Figure 2. ^{31}P NMR spectra of lipid-modified 5´-DMT-2´-dU-phosphoramidite **1**

Lipidated *anti*-cMet aptamer trCLN3-L4 and its non-binding mutant trCLN3.mut-L4

The *anti*-cMet aptamer trCLN3 is a 40 nucleotide DNA oligodeoxynucleotide that forms two intramolecular G-quadruplex structures within the G-rich segments (Supplementary Fig. 3). Filter retention assays with a ^{32}P-labeled variant showed that trCLN3 binds to cMet in nanomolar concentrations.[2] The binding affinity of trCLN3.mut, a control sequence with a guanine double-point mutation corresponding to G7 and G25 was further verified by filter retention assays. As almost no binding was observed for the two-point mutant control sequence, it was used as non-binding variant in further experiments.

Sequences:

trCLN3: 5'-TGGATG**G**TAGCTCGGTCGGGGT GG**G** TGGGTTGGCAAGTCT-3'

trCLN3.mut: 5'-TGGATG**A**TAGCTCGGTCGGGGT GG**A** TGGGTTGGCAAGTCT-3'

trCLN3-L4: 5'-LLLLTGGATG**G**TAGCTCGGTCGGGGT GG**G** TGGGTTGGCAAGTCT-3'

trCLN3.mut-L4: 5'-LLLLTGGATG**A**TAGCTCGGTCGGGGT GG**A** TGGGTTGGCAAGTCT-3'

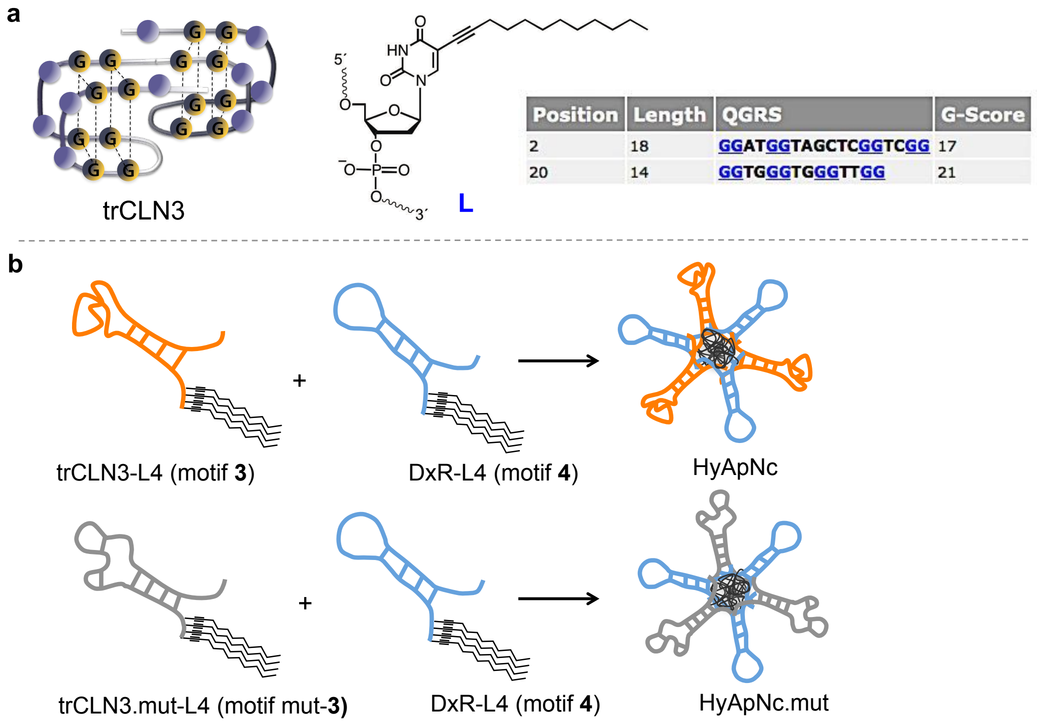

Supplementary Figure 3. Predicted secondary structures of aptamers trCLN3 and lipid-mediated self-assembly. **a** Two G-quadruplexes were predicted using GQRS Mapper.[3] **b** Schematic representation of the lipid-mediated self-assembly of cMet binding motif trCLN3-L4 (motif **3)** and doxorubicin binding motif **DxR**-L4 (motif **4)** forms HyApNc. A non-cMet-binding mutant trCLN3.mut-L4 (motif mut-**3**) was used instead of motif-**3**, resulting in a mutated nanoconstruct HyApNc.mut. For **DxR**-L4 motif see Fig. 3**a** and Supplementary Methods

Four C_{12}-lipid chains (L) were coupled to trCLN3 in a single process using a standard phosphoramidite solid-phase DNA synthesis protocol. trCLN3-L4 and its non-binding mutant trCLN3.mut-L4 with the 40 nucleotide sequence (Supplementary Fig. 3) both were synthesized in 200 nmol scale using an ABI 3400 DNA synthesizer. The lipid-modified uridine-phosphoramidite **1** (0.221 g) was dissolved in DNA-grade dichloromethane (2.7 mL) under Ar atmosphere to give a 0.1 M solution. The syntheses of trCLN3-L4 and trCLN3.mut-L4 were performed identically, except for the ODN sequences. After the last detritylation step

the lipidated uridine phosphoramidite **1** was coupled to the detritylated 5′-end of the oligonucleotide chain, using an optimized coupling procedure. Deprotection of phosphate groups and protected amino nucleobases as well as cleavage of the product from the solid support was carried out by incubation in a 50:50 (v/v) mixture of 30% NH_3 solution (400 µL) and methyl amine (400 µL) for 2 h at 55 °C. The solid support was then removed by filtering and was washed with an ethanol/water (50:50, v/v) mixture. The filtrate was concentrated under reduced pressure and dried.

Reversed-phase HPLC purification

Following deprotection and separation from the solid-support, the lipid-functionalized aptamers trCLN3-L4 & trCLN3.mut-L4 were purified by using reversed-phase high performance liquid chromatography (HPLC) on an Eclipse XBD C18 column using 0.1 M TEAAc (A) and acetonitrile (B) with a gradient of A/B = 98/2 → 35/65 in 30 min. The coupling yield of the labeling reaction was determined to be 31% trCLN3-L4 and 29% trCLN3.mut-L4, respectively, by integration of the peaks in the HPLC chromatogram (Supplementary Fig. 4). The purified lipid-modified ODN fraction was concentrated using a freeze-dryer. ODN concentrations were determined by UV absorbance using extinction coefficients at λ = 260 nm. Finally, the identity of the oligonucleotides was confirmed by ESI-mass spectrometry (Supplementary Fig. 5).

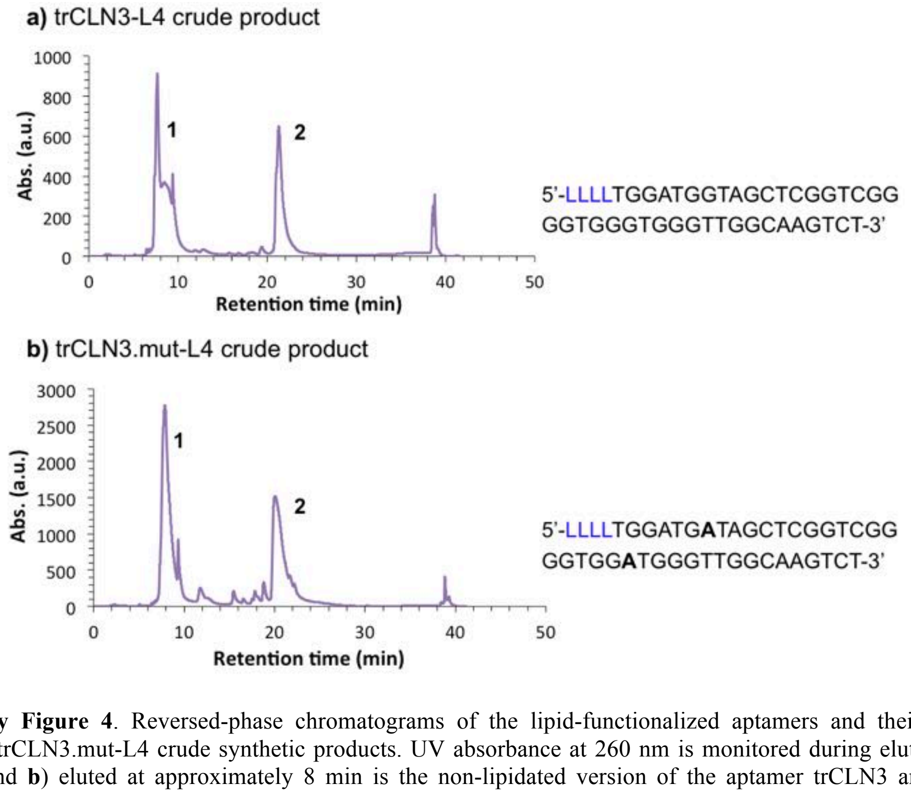

Supplementary Figure 4. Reversed-phase chromatograms of the lipid-functionalized aptamers and their sequences. **a** trCLN3-L4, **b** trCLN3.mut-L4 crude synthetic products. UV absorbance at 260 nm is monitored during elution. Fraction 1 (shown in **a** and **b**) eluted at approximately 8 min is the non-lipidated version of the aptamer trCLN3 and trCLN3.mut whereas fraction 2 eluted at approximately 22 min and corresponds to the lipid-functionalized aptamer

ESI Mass Spectrometry

The molecular masses of *anti*-cMet aptamer trCLN3 and its lipid-functionalized derivatives were further analyzed by ESI-LCMS in negative ion mode using a Bruker Esquire 6,000 ion-trap MS system with an electrospray ionization source coupled to an Agilent 1100 series HPLC system modified with a ZORBAX SB-18 analytical column (2.1×50mm). The ESI mass spectra of the purified trCLN3 aptamer and its lipid-functionalized conjugates are shown in Supplementary Fig. 5. An elution buffer (10 mM TEA + 100 mM HFIP) in combination with linear gradients of acetonitrile from 0% to 80% in 30 min was used as mobile phase for analysis. The *m*/*z* ratio is calculated by deconvolution of the ionic fragments.

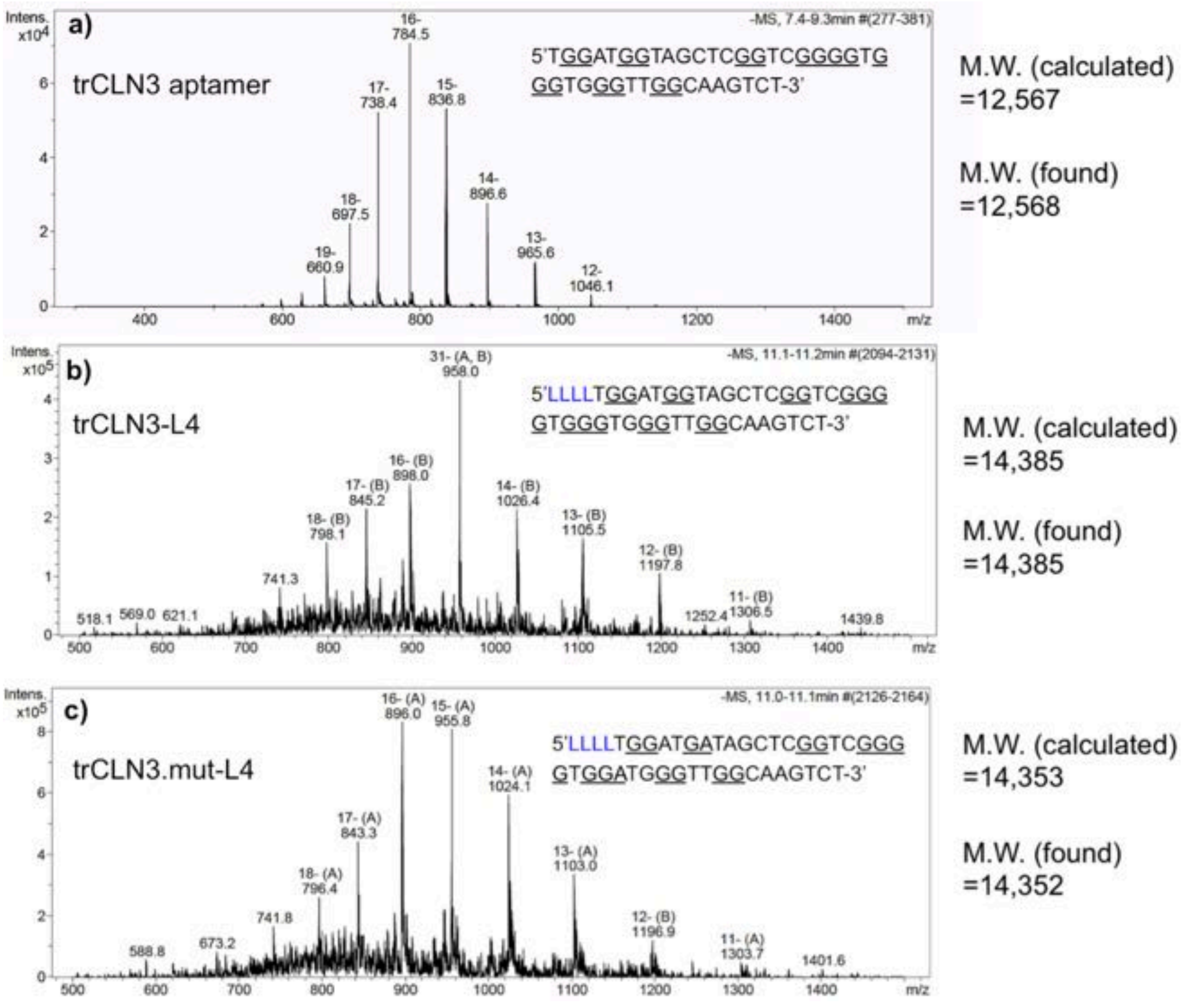

Supplementary Figure 5. ESI mass spectra of the HPLC-purified aptamer motifs. **a** Native trCLN3 aptamer **b** Lipid-functionalized derivative trCLN3-L4. **c** Lipid-functionalized two point mutant trCLN3.mut-L4. The corresponding expected and observed molecular masses of the aptamers are shown at the right of the individual ESI mass spectra

Critical micelle concentrations via FRET studies

The critical micelle concentration (CMC) value of the trCLN3-L4 aggregates was determined by intermolecular Förster resonance energy transfer (FRET) experiments using a FRET pair of 6Fam and Atto647N both attached to the 5´-end of the trCLN3-L4 motif **3**. The FRET labels were attached at the 5´-end in immediate proximity to the lipid-modifications to ensure that intermolecular FRET effects report the formation of micellar nanoconstructs at a concentration above the critical micelle concentration.

In the FRET experiment, a series of nanoconstructs was self-assembled by mixing 6Fam- and Atto647N-labeled motif **3** in 1:1 ratios in a concentration range between 0.035-10 µM (Supplementary Table 1). The solutions were incubated at 70 °C for 10 min in the dark and slowly cooled down to room temperature overnight at a rate of 1 °C per 10 min. The mixtures were transferred into a 384-well plate and the FRET effect was monitored at room temperature by using an excitation wave length of λ_{ex} = 480 nm and an emission wavelength of λ_{em} = 669 nm using an EnSpire® Multimode Plate Reader (PerkinElmer).

Supplementary Table 1. Concentrations of 6-Fam- and Atto647N-labeled motifs **3** mixed in 1:1 ratios to form mixed micellar nanoconstructs.

Exp. No.	6fam-**3** [µM]	atto647-**3** [µM]	Volume (µL)	Ratio 6fam: atto647	I_{669}	I_{520}	I_{669}/I_{520}
01	10.0	10.0	20	1:1	17481	4152	4.21
02	5.0	5.0	20	1:1	10876	2254	4.82
03	2.5	2.5	20	1:1	5176	1062	4.87
04	1.0	1.0	20	1:1	1526	501	3.04
05	0.5	0.5	20	1:1	585	434	1.35
06	0.25	0.25	20	1:1	71	139	0.51
07	0.125	0.125	20	1:1	97	114	0.85
08	0.07	0.07	20	1:1	16	78	0.21
09	0.035	0.035	20	1:1	6	126	0.05

The intensity signals were collected for both FRET channels at λ_{em} = 669 nm for the acceptor channel and that of donor channel at λ_{em} = 520 nm. The concentration dependent intensity ratios (I_{669}/I_{520}) were plotted as a logarithmic function depending on the trCLN3-L4 concentration. The CMC value was determined from the intersection of the lower horizontal asymptote of the sigmoidal curve with the tangent at the inflection point corresponding to the minimum trCLN3-L4 concentration required for formation of stable micelles in aqueous medium. The CMCs of trCLN3-L4 aggregate was determined to be 300 nM (~ 0.005 mg mL^{-1}).

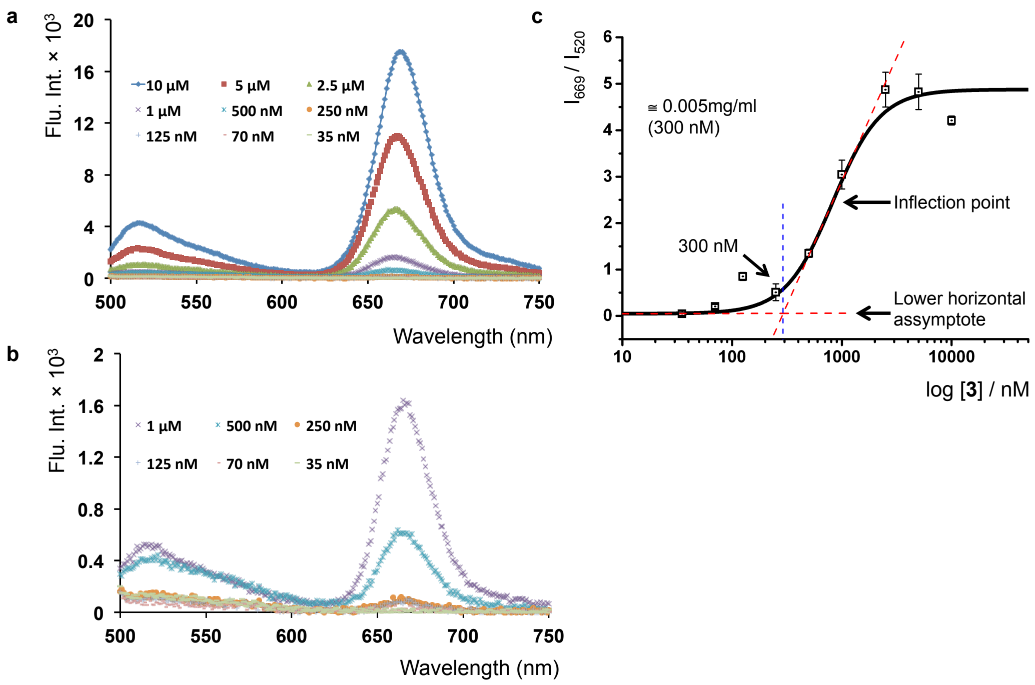

Supplementary Figure 6. Critical Micelle Concentrations (CMC) determination using 6Fam- and Atto647N- labeled motif **3** as FRET pairs in 1:1 ratio in a varied concentration range. **a** Fluorescence emission spectra (λ_{ex} = 480 nm; λ_{em} = 669 nm) for FRET assembled 6Fam-**3**/ Atto647N-**3** nanoconstructs. **b** Magnification of the emission spectra in 1 µM – 35 nM range. **c** The change of intensity ratio I_{669}/I_{520} at different motif-**3** concentrations (error bars: n = 3, mean ± S. E.M.)

Critical micelle concentrations from pyrene fluorescence

Critical micelle concentration (CMC) value of the trCLN3-L4 motif was further confirmed by internalizing pyrene into the hydrophobic-lipid core of the micellar aggregate followed by measuring the fluorescence of pyrene-loaded trCLN3-L4 nanoconstructs at different concentrations. For this experiment a fixed amount of pyrene in acetone was transferred to an empty tube and acetone was allowed to evaporate in the dark at 45 °C for 30 min using an Eppendorf concentrator. trCLN3-L4 solutions in the concentration range between 0.0005-0.5 mg mL^{-1} was then added to yield a final pyrene concentration fixed at 100 µM for all reactions (Supplementary Table 2). The solutions were incubated at 90 °C for 10 min in the dark and slowly cooled down to room temperature overnight at a rate of 1 °C per 10 min in order to internalize pyrene into the hydrophobic lipid core. The pyrene-loaded trCLN3-L4 nanoconstructs were transferred into a 384-multi well plate and the fluorescence emission spectrum of each well was recorded at room temperature by using an excitation wave length of 339 nm in an EnSpire® Multimode Plate Reader (PerkinElmer).

Supplementary Table 2. Concentrations for trCLN3-L4 **3** micelles and pyrene in a fixed reaction volume of 50 µL used for CMC determination of trCLN3-L4 aggregated nanoconstructs.

Exp. No.	trCLN3-L4 **3** [mg/mL]	trCLN3-L4 **3** [µM]	Pyrene[µM]	Volume [µL]	I_{475}	I_{373}	I_{475}/I_{373}
01	0.5	35	100	50	174827	22321	7.83
02	0.25	17.4	100	50	130337	18886	6.90
03	0.1	7.0	100	50	90719	17675	5.13
04	0.05	3.5	100	50	60458	12887	4.69
05	0.025	1.75	100	50	47267	18925	2.49
06	0.01	0.7	100	50	41638	26517	1.57
07	0.005	0.35	100	50	40004	20218	1.98
08	0.0025	0.175	100	50	14658	20188	0.72
09	0.001	0.07	100	50	4435	19581	0.23
10	0.0005	0.035	100	50	2751	13370	0.20

In close proximity, two pyrene molecules form an excimer that emits fluorescence at a longer wavelength compared to the monomer emission. The formed excimer is a dimeric complex where one molecule exists in an excited state and the other molecule in a ground state. Monomer emission of pyrene occurs within a range of 360-400 nm whereas the excimer emission is obtained within the wavelength limit of 465-500 nm. The critical micelle concentration was determined by the distinguishable pyrene excimer fluorescence of the corresponding DNA concentration.[4]

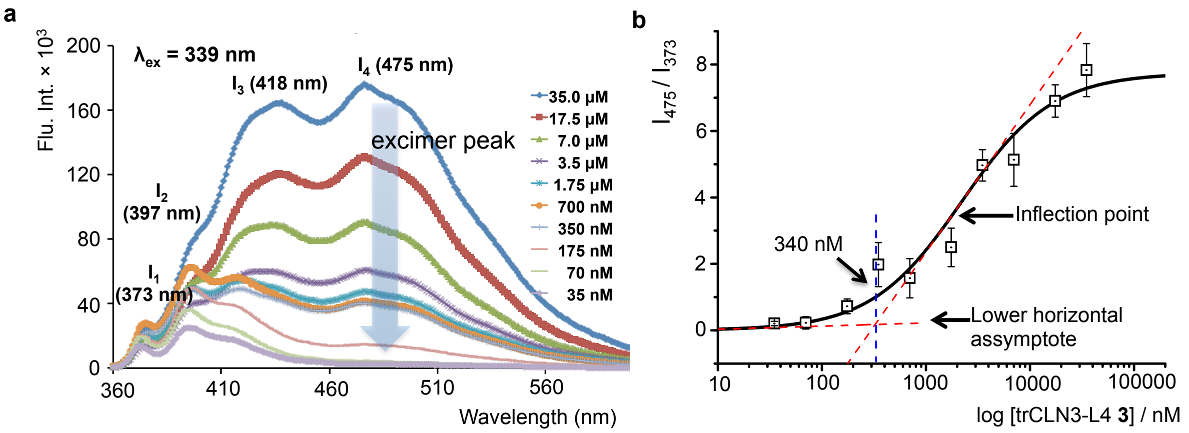

Supplementary Figure 7. CMC determination from the fluorescence of the pyrene probes incorporated to the hydrophobic lipid core of trCLN3-L4 aptameric nanoconstructs. **a** Fluorescence emission spectra (λ_{ex} = 339 nm) of pyrene-loaded trCLN3-L4 nanoconstructs at a fixed pyrene concentration of 100 µM and different trCLN3-L4 concentrations. **b** Variations of the intensity ratios I_{475}/I_{373} as a function of trCLN3-L4 **3** concentrations (error bars: n = 2, mean ± S. E.M.)

TEM analysis of trCLN3-L4 nanoconstructs

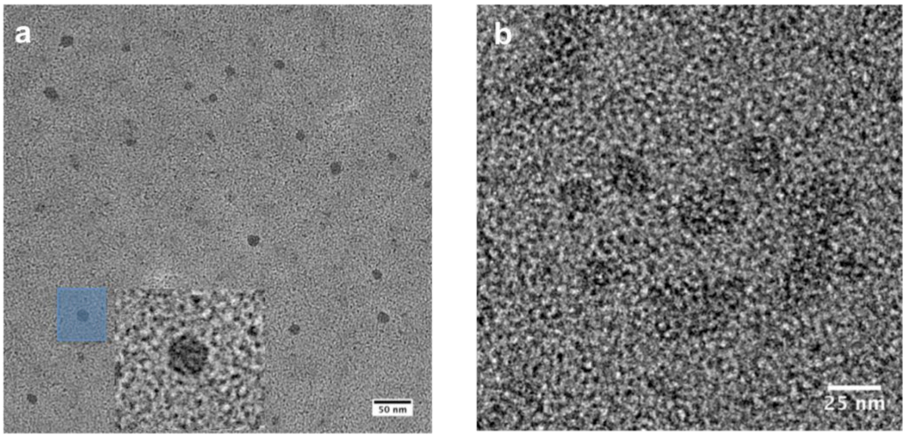

Supplementary Figure 8. TEM micrographs of the self-assembled trCLN3-L4 nanoconstructs with uranyl formiate staining. **a** Scale bar: black; 50 nm and **b** Scale bar: white; 25 nm. Inset: 5× zoom image of the same region

Influence of lipid tails on trCLN3 binding to cMet

To test the effect of lipid-modification on trCLN3 binding properties, we determined IC_{50} values for each trCLN3 derivative by a competitive filter retention assay in which varying concentrations of unlabeled 5´-(1-dodecynyl)-functionalized trCLN3 aptamers competed with constant amounts of γ-^{32}P-labeled trCLN3 in binding to cMet. Two control experiments were also performed using unlabeled trCLN3 and its two point mutant trCLN3.mut as competitors.

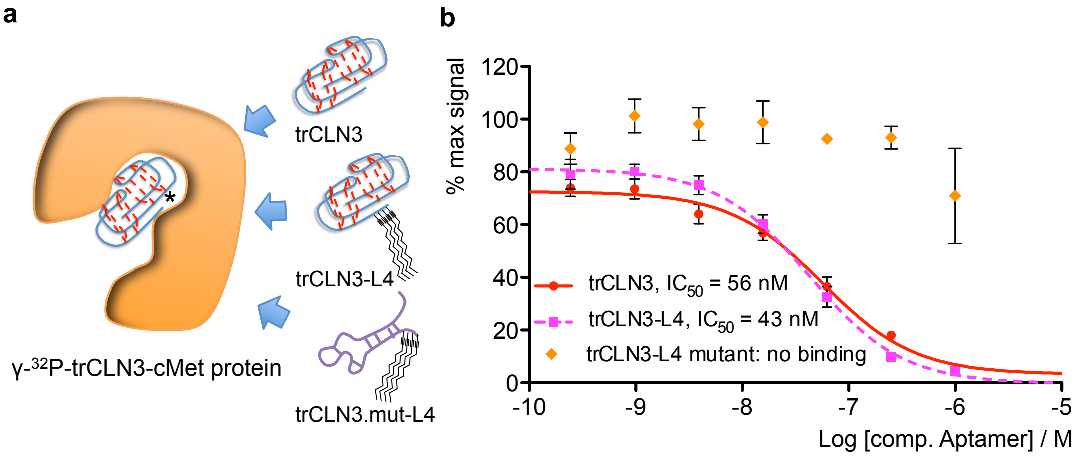

Supplementary Figure 9. Competitive filter retention assay. **a** Schematic of lipid-functionalized trCLN3 derivatives in varying concentrations competed with constant amounts of radiolabeled trCLN3 in binding to the target cMet. **b** Binding curves of trCLN3 (●), trCLN3-L4 (■), and trCLN3.mut-L4 (◆) to human cMet competing against γ-^{32}P-trCLN3 displaying the percentage of the maximum signal as a function of the amount of competing aptamer in a concentration range between10^{-10} to 10^{-6} (error bars: $n = 2$, mean ± S.D.)

First, the trCLN3 motif was 5´-end-labeled with γ-^{32}P ATP using T4 polynucleotide kinase. An aliquot of 20 µL solution containing 50 pmol trCLN3, 6.7 pmol γ -^{32}P ATP and 20 U T4 polynucleotide kinase in 1× polynucleotide kinase buffer (New England Biolabs) was incubated at 37 °C for 45 min, followed by removal of unreacted γ-^{32}P ATP using an Illustra

G-25 microspin column (GE Healthcare, München, Germany). The purity of the radiolabeled aptamer was confirmed using a 10% PAGE-gel.

To determine the affinity, ~25 fmol of radiolabeled aptamer was incubated with a cMet concentration of ~50 nM together with varying concentrations (1 µM – 25 pM) of unlabeled competitor for 30 min at 37 °C in 25 µL of buffer containing 0.1 mg mL^{-1} *E.coli* tRNA (Roche, Mannheim, Germany), 0.25 mg mL^{-1} BSA, 2 mM $MgCl_2$ in 1× PBS, pH 7.4. The aptamer protein complexes were captured on a Protran nitrocellulose membrane (GE Healthcare) that was pre-incubated in 0.4 M KOH for 10 min, followed by washing with 1× PBS containing 2 mM $MgCl_2$, pH 7.4. After addition of the aptamer-protein solution, the filter was washed 4 times with 1× PBS containing 2 mM $MgCl_2$ using vacuum filtration. Residual radioactivity due to cMet bound labeled aptamers was quantified using Fujifilm Fla-3000 PhosphorImager and AIDA software. The curves were fitted with GraphPadPrism 3.02 plotting non-linear regression curve and the IC_{50} values were calculated assuming a competition for a single binding site.

Serum stability of trCLN3 aptamer and their lipid-functionalized derivatives in fetal calf serum

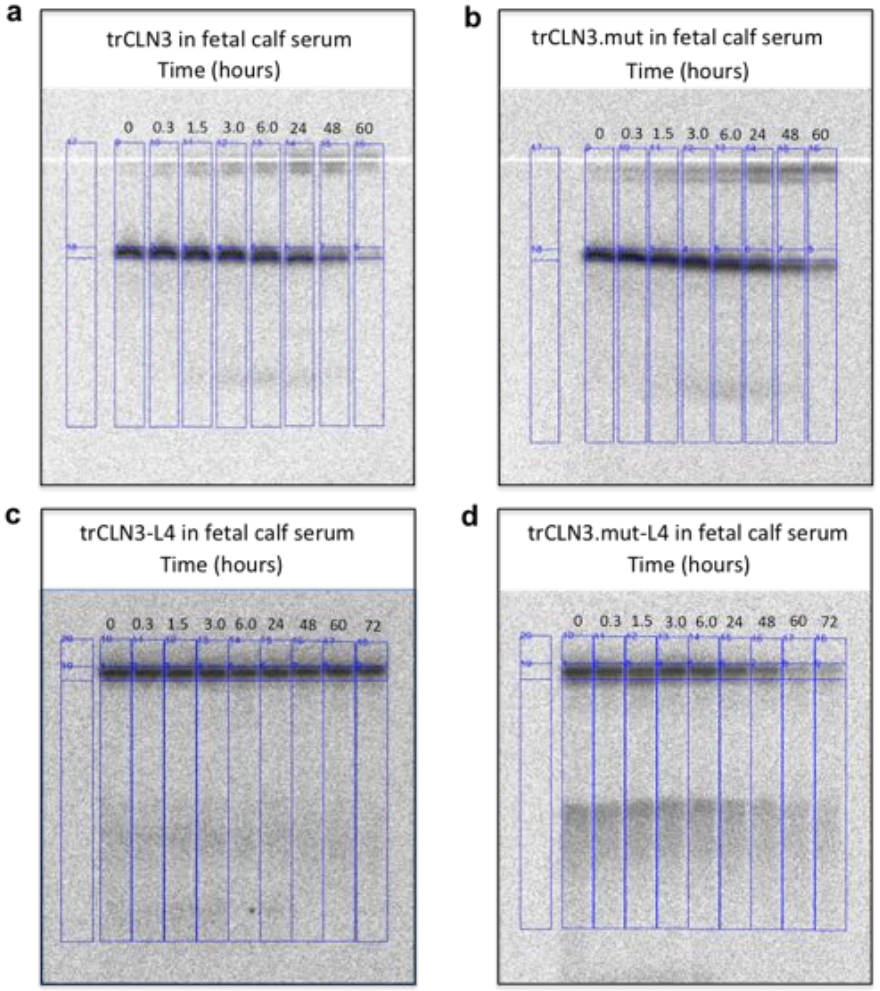

Supplementary Figure 10: Time dependent serum stability of trCLN3 aptamer and its lipid-modified derivatives in 90% PBS buffered FCS (FCS/10× PBS = 90/10) by PAGE. **a, b** PAGE-gel-images of the unmodified trCLN3 and trCLN3.mut aptamer. **c, d** Corresponding lipid-functionalized motifs trCLN3-L4 and trCLN3.mut-L4 after incubation in FCS (cropped images are produced in Fig. 2a)

Serum stability of trCLN3 aptamer and their lipid-functionalized derivatives in human blood serum

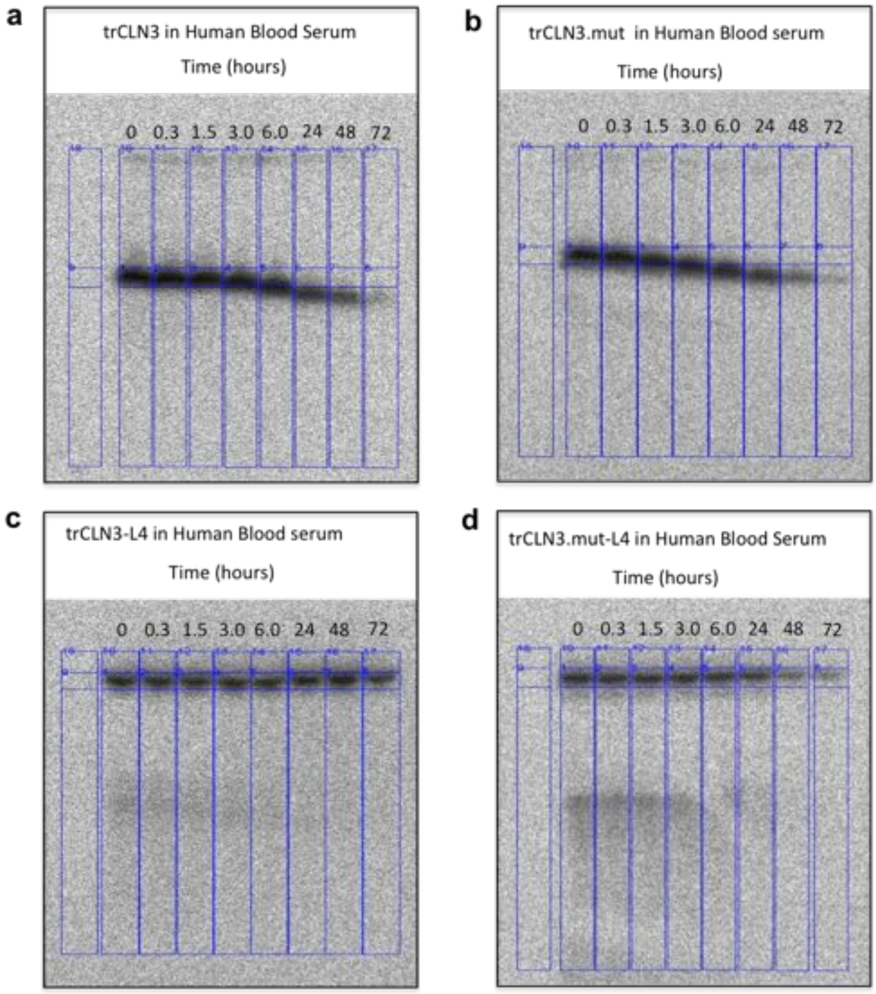

Supplementary Figure 11: Time dependent serum stability of trCLN3 aptamer and its lipid-modified derivatives in 90% PBS buffered HBS (HBS/10× PBS = 90/10) by PAGE. **a, b** PAGE-gel-images of the unmodified trCLN3 and trCLN3.mut aptamer. **c, d** Corresponding lipid-functionalized motifs trCLN3-L4 and trCLN3.mut-L4 after incubation in HBS (cropped images are shown in Fig. 2b)

Synthesis of DMTr-protected 2´,6´-dimethylazobenzene phosphoramidite

DMTr-protected phosphoramidite carrying a 2´,6´-dimethylazobenzene moiety on a D-threoninol backbone (**2**) was synthesized as reported elsewhere.[5]

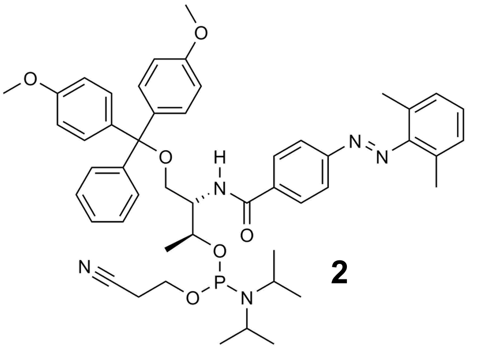

2

DMTr-protected phosphoramidite carrying a 2´,6´-dimethylazobenzene (**2**)

Characterization data: TLC (Cy-hexane: EtOAc, 50:50 v/v + 3% Et_3N): $R_f = 0.65$; ^{31}P NMR (162 MHz, $CDCl_3$): δ 148.72 (s), 149.16 (s); HRMS (*m/z*): $[M+Na]^+$ calcd. for $C_{49}H_{58}N_5O_6PNa$, 866.4017; found, 866.4011

Synthesis of doxorubicin-carrying **DxR**-L4 motif

DMAB-phosphoramidite and lipid-phosphoramidite were introduced as a photo-trigger and lipid-tails to the doxorubicin carrying **DxR**-L4 motif by solid phase DNA-synthesis. The motif consists of a 37-nucleotide DNA sequence with 4 DMAB moieties introduced into the sequence and four lipid-tails attached to the 5´-end. The resulting purified doxorubicin-carrying **DxR**-L4 (**4**) motif (Fig. 3a) was analyzed by ESI-LCMS mass spectrometry.

ESI Mass Spectrometry of **DxR**-L4 motif

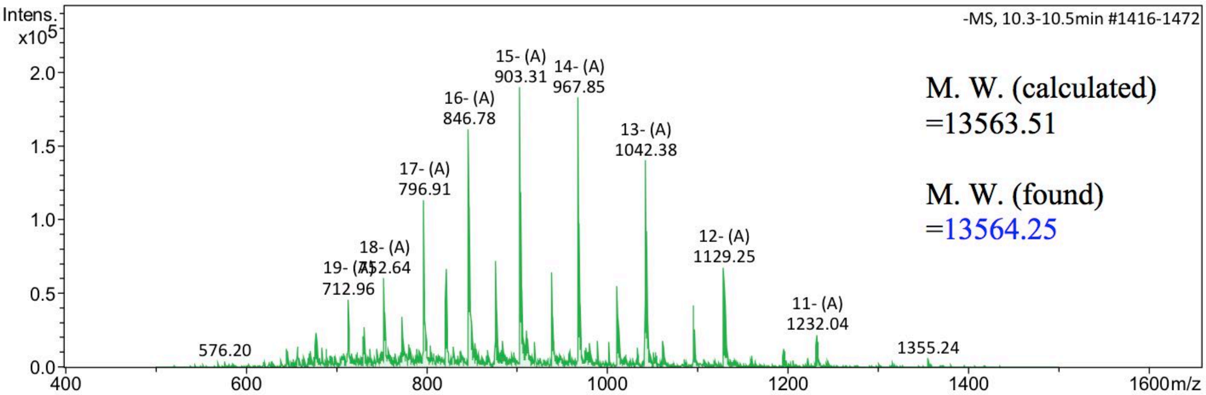

Supplementary Figure 12. ESI mass spectra of the doxorubicin carrying **DxR**-L4 motif **4**. The corresponding expected and observed molecular masses of the aptamers are shown at the side of the ESI mass spectrum

The molecular mass of lipid-functionalized **DxR**-L4 motif **4** was analyzed by ESI-LCMS in negative ion mode (Bruker Esquire 6,000 ion-trap MS system with an electrospray ionization

source coupled to an Agilent 1100 series). The ESI mass spectrum of the purified lipid-functionalized **DxR**-L4 motif **4** is shown in Supplementary Figure 12. Deconvolution of the ionic fragments leads to a measured total mass of MW_{meas} = 13564.25 corresponding to the target oligonucleotide with the calculated mass of MW_{calc} = 13563.51.

Reversible switching of DMAB functionalized DxR-L4 motif

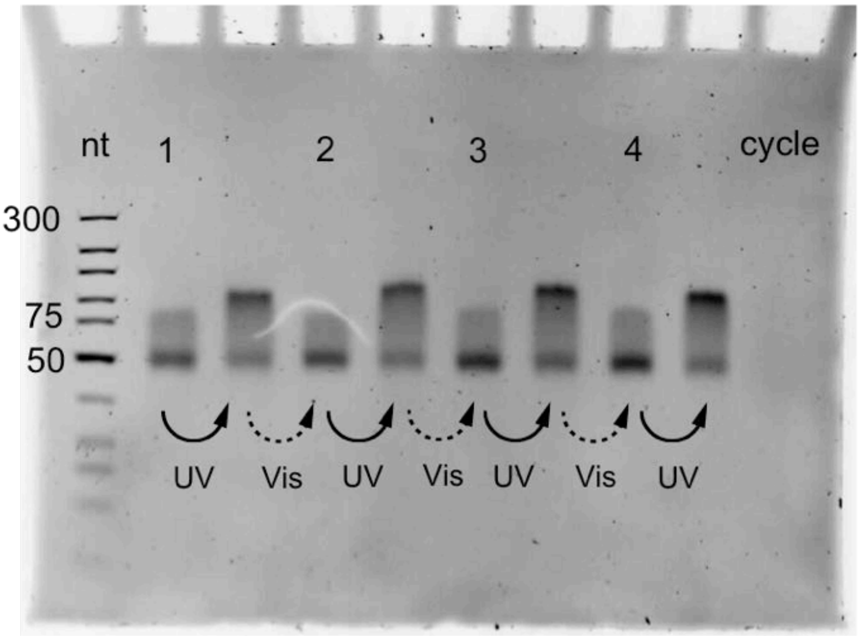

Supplementary Figure 13. PAGE analysis of reversible switching of 2',6'-dimethylazobenzene functionalized **DxR**-L4 motif **4** (cropped image shown in Fig. 3d)

Doxorubicin intercalation to motif **4** and purification

A fixed amount of motif **4** (5 µM) was added to a 10-fold excess of **DxR** in buffer (1× PBS + 1 mM $MgCl_2$) and incubated for 12 h at room temperature. The motif **4-DxR** complex was transferred to an Amicon®Ultra-0.5 centrifugal filter column with 3K molecular weight cutoff and excess of free doxorubicin was removed by three times consecutive centrifugation at 14,000 g for 10 min at room temperature while adding fresh buffer at each centrifugation step. After each centrifugation step, a UV/Vis- spectrum of the supernatant and flow through washing was recorded and a reduction in doxorubicin absorbance further confirmed the successive removal of excess doxorubicin through repeated washing.

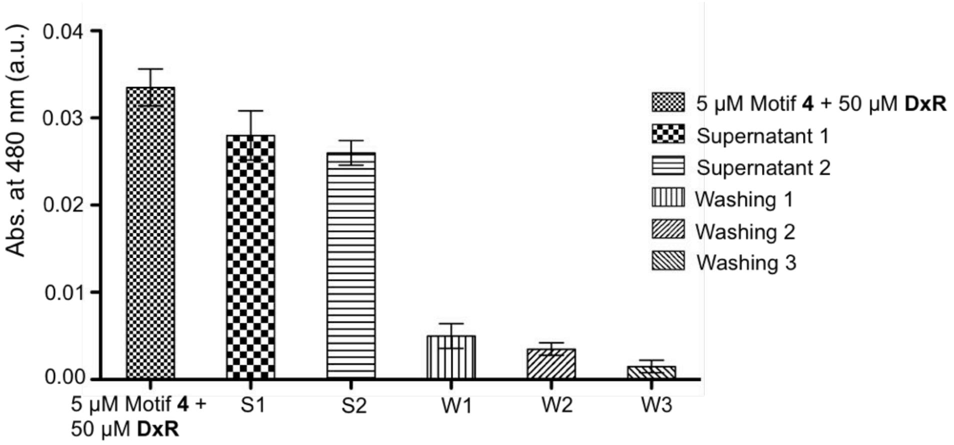

Supplementary Figure 14. UV/Vis- absorbance of the corresponding supernatants and flow through washings after each centrifugation step (error bars: $n = 2$, mean ± S.D.)

Quantification of the doxorubicin release from loaded motif **4** by HPLC assay

The release of **DxR** bound to motif **4** was analyzed by ion-exchange chromatography on a TSKgel DEAE-NPR Guard 2.5 µm 4.6×5 mm column. A mobile phase of 1× PBS buffer + 5% ACN (mobile phase A) and 1× PBS buffer + 1M NaCl + 5% ACN (mobile phase B) were used with a gradient of A/B = 100/0 → 0/100 over 20 min. A fully encapsulated motif **4-DxR** complex was incubated at 37 °C in 1× PBS buffer. For each measurement an aliquot of 20 µL sample solutions was removed after the indicated time interval and irradiated with 365 nm light for 5 min. Samples that were not irradiated were used as controls. Following the UV exposure, the samples were extracted twice with phenol/$CHCl_3$ and twice with $CHCl_3$, which removed the excess **DxR** released by photoirradiation. It was already reported that the phenol/$CHCl_3$ (1:1) washing removes unbound excess doxorubicin after intercalation into DNA duplexes without removing the intercalated doxorubicin.[6] Afterwards, 10 µL of each sample was injected and the remaining **DxR** bound to motif **4** was quantified by recording the fluorescence at 590 nm (λ_{ex}= 490 nm) using a flow-through fluorescence detector attached to the HPLC.

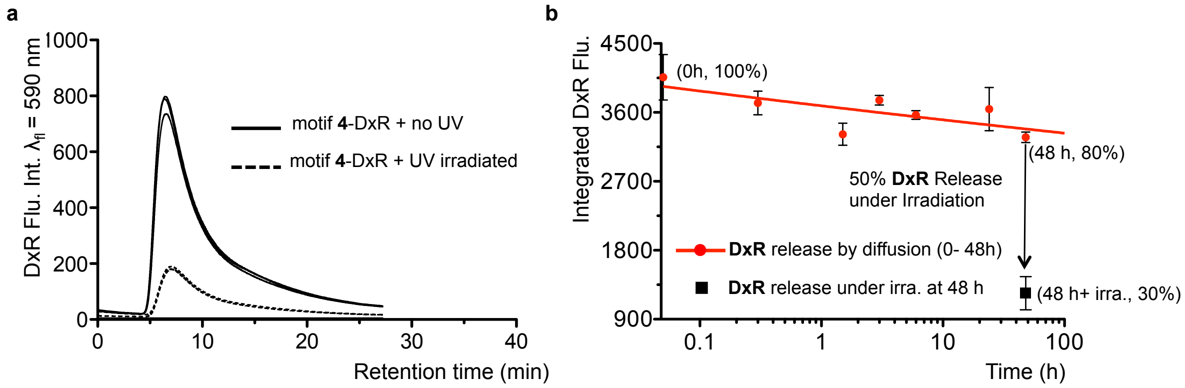

Supplementary Figure 15. Photocontrolled and thermal release of remaining **DxR** bound to motif **4** after removing unbound excess **DxR** from the solution by phenol/$CHCl_3$ (ref. 6) monitored by HPLC assay. **a** HPLC chromatogram of the motif **4-DxR** complex with and without UV exposure (dotted vs. solid line). The release curves of **DxR** were obtained by measuring the fluorescence at 590 nm using a fluorescence detector attached to the HPLC. After 5 min of UV irradiation, motif **4-DxR** complex displayed a 63% reduction in fluorescence compared to non-irradiated samples. **b** Release of **DxR** bound to motif **4** incubated at 37 °C solely through self-diffusion at different times over 48 h (percentage of **DxR** bound to motif **4** at different incubation time are shown in brackets). 0 h sample represents 100% **DxR** bound to motif **4**. A 20% reduction in fluorescence was observed for the motif **4-DxR** complex which was incubated for 48 h (●). The 48 h sample was then exposed to UV light for 5 min, which further reduced the fluorescence by 50% (■) (error bars: n = 2, mean ± S.D.)

FRET efficiency of assembled particles with both **D** (a550-**DxR**-L4) and **A** (a647N-trCLN3-L4) motifs

We performed steady-state fluorescence measurements on a Fluoromax 3 fluorometer [Horiba Jobin-Yvon] at 25 °C. Excitation wavelengths were at 554 nm (excitation of Atto550) and 644 nm (excitation of Atto647N), respectively. The entrance and exit slits were set to 5 nm, and integration time was set to 0.5 s. Apparent experimental FRET efficiencies were calculated using the direct method through

$$E = \left(\frac{I_A}{q_A}\right)\left(\frac{I_A}{q_A} + \frac{I_D}{q_D}\right)^{-1}$$

where I_A is the acceptor peak fluorescence intensity after donor excitation from which contribution from donor fluorescence was subtracted, I_D is the donor peak fluorescence intensity after donors excitation, and the values for q_A (0.65) and q_D (0.8) are quantum yields of Atto647N and Atto550 dyes, respectively. The calculation of FRET efficiency for the atto dyes are not yet well studied and our calculation is indicative and a good approximation of changes in the distances. This calculation does not provide absolute values of distance between the dyes, however, it is an effective way to determine relative changes in distance between the fluorophores. Nanoconstructs assembled with motifs Atto647N-**3** and Atto550-**4** (HyApNc) yielded a FRET efficiency of 92% as compared to 27% where both motifs **3** and **4** lack the lipid modifications (**F6** vs. **F5**). When a non-cMet-binding Atto647N-labeled mutant trCLN3-L4 motif (Atto647N.mut-**3)** was used instead of Atto647N-**3,** the resulting mutated nanoconstruct HyApNc.mut yielded a similar FRET efficiency (97%) as shown by HyApNc (**F7** vs. **F6**). These results provide clear evidence that both motifs properly assemble in presence of 5'-lipid modification to form hybrid nanoconstructs as compared to the non-lipidated motifs.

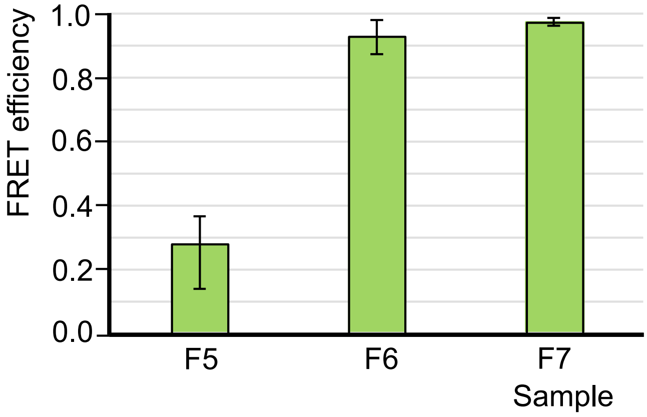

Supplementary Figure 16. FRET efficiency comparision for (λ_{ex} = 554 nm; λ_{em} = 669 nm) HyApNc consisting of motifs Atto550-**4** and Atto647N-**3** without (~27%, **F5)** and with lipid tail (92%, **F6**). Mutated nanoconstructs (HyApNc.mut) consisting of Atto647N.mut-**3** motif and Atto550-**4** exhibited similar FRET effect as shown by HyApNc (~97%, **F7**) (error bars: n = 3, mean ± S.D.)

Stability of HyApNc micellar nanoconstructs in presence of Human Blood Serum (HBS) and Bovine Serum Albumin (BSA)

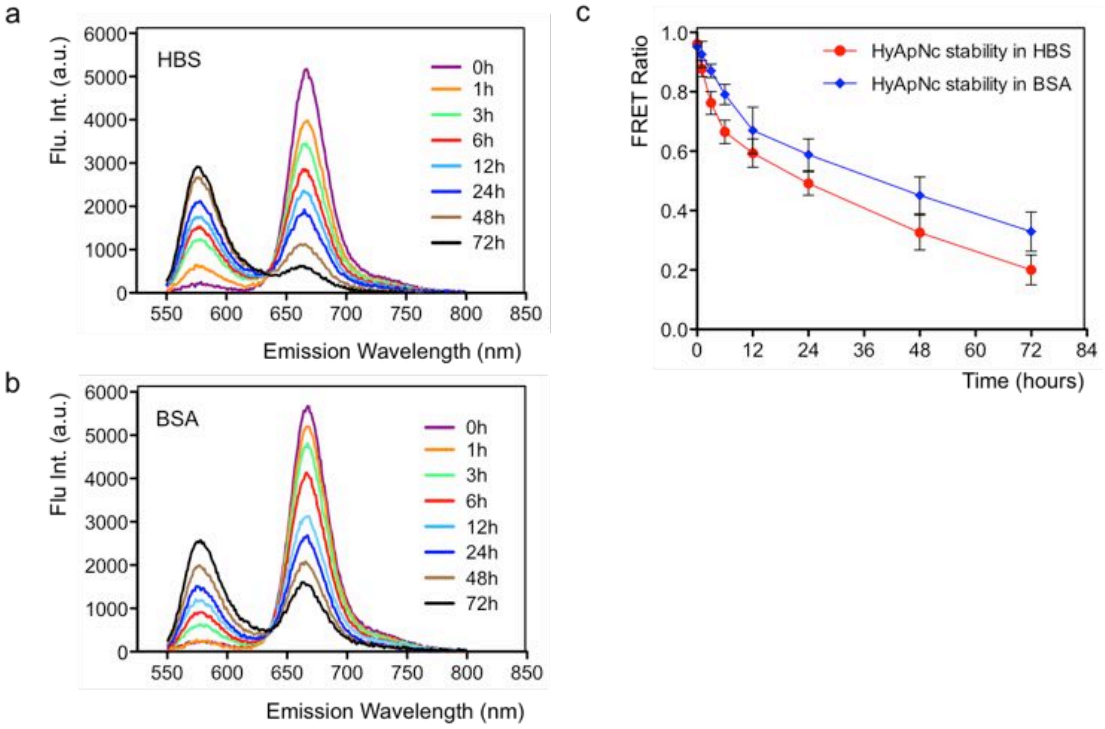

Supplementary Figure 17. Time-resolved FRET spectra of dual-labeled micellar nanoconstructs. **a** Integrity of HyApNc in 95% human blood serum (HBS). **b** Integrity of HyApNc in 1 mM bovine serum albumin solution (BSA). **c** Time traces of the FRET ratio = I_{669} $(I_{669} + I_{576})^{-1}$, in human blood serum (●) and in solutions of BSA (◆) (error bars: $n = 2$, mean ± S.D.)

The integrity of the micellar nanoconstruts HyApNc was tested in a FRET assay[7, 8] in presence of human blood serum (HBS) and in bovine serum albumin (BSA) solution. A suitable FRET pair Atto-647N-**3** as the acceptor and Atto550-**4** as the donor was used to assess the stability of micelles in the presence of 95% HBS and 1 mM BSA solution. In a FRET experiment, 2 µM of HyApNc containing the FRET pair (Atto647N-**3** & Atto550-**4**) in 1:1 ratios were incubated with 95% HBS and 1mM BSA solutions separately at 37 °C. For each measurement an aliquot of 20 µL samples were taken after indicated time intervals of 0, 1, 3, 6, 24, 48 and 72 h respectively, transferred into a 384-well plate and the time-resolved fluorescence spectra of FRET pairs were measured by using an excitation wave length of λ_{ex} = 535 nm and an emission wavelength spectrum between λ = 550 nm and λ = 800 nm was recorded using an EnSpire® Multimode Plate Reader (PerkinElmer). The FRET ratio was calculated by using the equation FRET ratio = I_{669} $(I_{669} + I_{576})^{-1}$, which yields the relative stability of the micelles. The approximate half-life of the HyApNc was estimated to be ($t_{1/2}$) of 14 h in 95% human blood serum and 18 h in 1 mM BSA solution respectively. The FRET ratios show a decrease in the FRET efficiency over time indicating that the micellar nanoconstcuts not withstand in presence of high serum proteins for very longer period of time and gradually disassembled over a period of 72 h.

Atto647N-3 internalization at concentrations above or below CMC

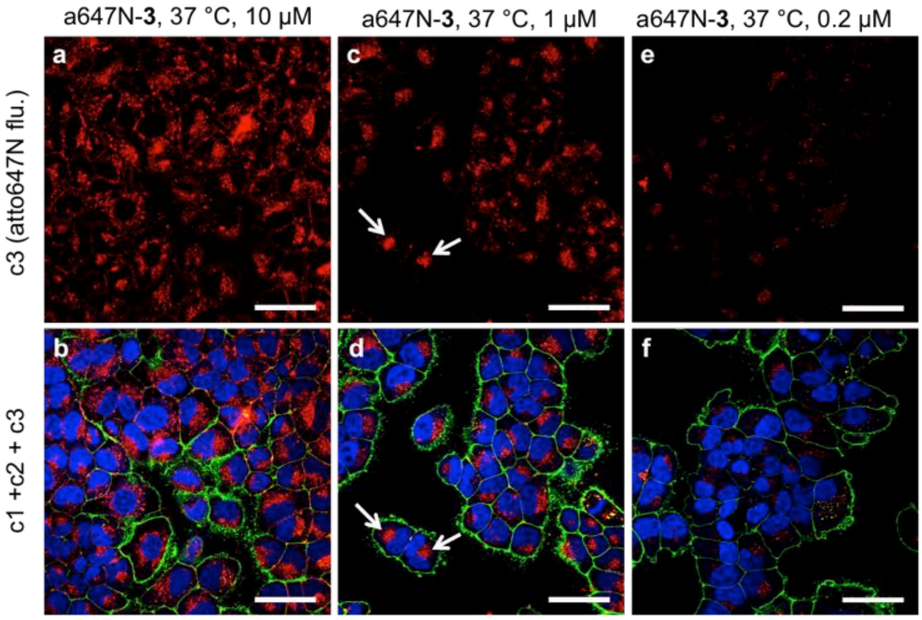

Supplementary Figure 18. Confocal microscopy analysis of NCI-H1838 cells incubated with Atto647N labeled trCLN3-L4 nanoconstructs at 37 °C. **a**, **b** Cells incubated with 10 µM A647N-**3** (**a**; red; c3, **b**; overlay: c1+c2+c3). **c, d** Cells incubated with 1 µM A647N-**3** (**c**; red; c3, **d**; overlay: c1+c2+c3). **e**, **f** Cells incubated with 0.2 µM A647N-**3** (**e**; red; c3, **f**; overlay: c1+c2+c3). Cells were membrane stained with Alexa488-WGA (green; c2), nuclei were stained with Hoechst 33342 (blue; c1) and analyzed for Atto647N-**3** uptake (red; c3). The arrow shows a punctuated fluorescent pattern in Figure **c**, **d**, which indicates that the A647N-**3** nanoconstructs might localize in the endosomes. Scale bar: **a**–**f**, 50 µm

Flow cytometry analysis

For analysis of trCLN3 internalization using flow cytometry, approximately 1 × 10^5 NCI-H1838 cells/well were seeded in a 24-well plate and incubated for 24 h at 37 °C. After 24 h of incubation, the cells were washed with 200 µL of 1× PBS and then incubated with 200 µL of 1 µM Atto647N labeled aptamer motifs A647N-**3** at 37 °C, A647N-**3** at 4 °C, A647N.mut-**3** at 37 °C, and A647N-trCLN3$_{w/oL4}$ at 37 °C, respectively, for 2 h. The cell medium was removed and the cells were detached from the plates using trypsin-EDTA and transferred to FACS tubes. The cells were then washed twice by centrifugation with 0.5 mL buffer and the cell pellets were resuspended in 100 µL of 1× PBS buffer and subjected to flow cytometric analysis using a BD FACS Canto™ II Flow Cytometer (BD Biosciences). Fluorescence emissions from Atto-647 labeled aptamer motifs (data presented in Fig. 5i) were collected with a 660/20-nm band-pass filter. A minimum detection of 10,000 events were collected and analyzed with the FlowJo software program.

For flow cytometry analysis of HyApNc-mediated **DxR** uptake, the H1838 cells (1 × 10^5 cells/well) were seeded for 24 h at 37 °C. The cells were washed with 1× PBS (200 µL) and subsequently treated with free **DxR** (as control), or targeted nanoconstructs HyApNc-**DxR**, or mutated non-targeted nanoconstructs HyApNc.mut-**DxR**, or HyApNc$_{w/oAz}$-**DxR** with a final **DxR** concentration of 8 µM in the culture medium. The plates were then incubated for 2 h at 37 °C. Afterwards, the cells were detached from the plates by trypsinization and transferred to

FACS tubes. The cells were then washed twice by centrifugation with 0.5 mL buffer. Afterwards, the cell pellets were resuspended in 100 µL 1× PBS buffer and either irradiated with UV light for 5 min (λ=365 nm, 350 mW cm^{-2}) or not irradiated before subjected to FACS analysis. Fluorescence emissions of the internalized **DxR** were recorded with a 585/42-nm band-pass filter.

FRET analysis of the intracellular integrity of the HyApNc

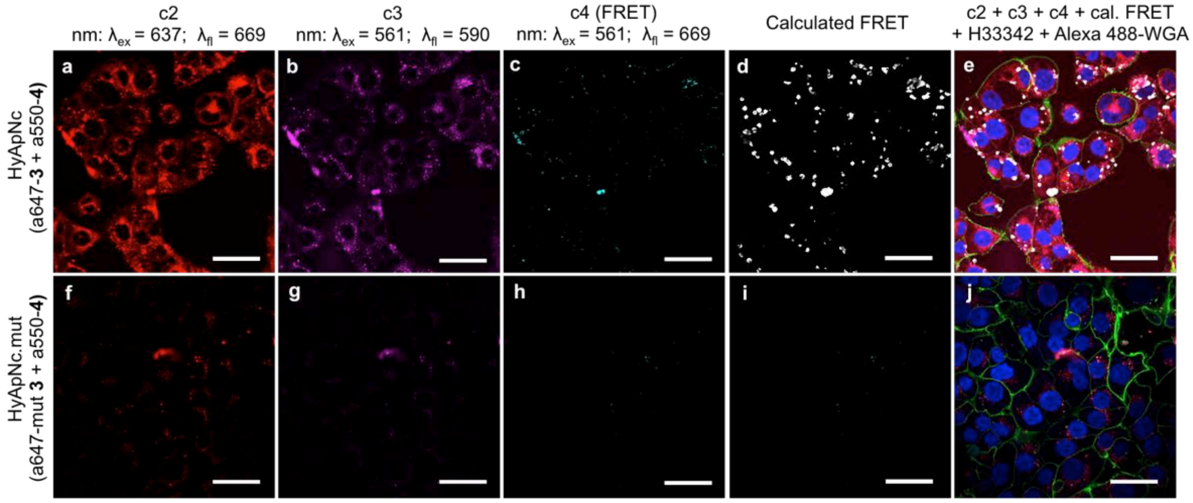

Supplementary Figure 19. Intracellular FRET analysis of H1838 cells incubated with dual-labeled HyApNc nanoconstructs. **a**–**e** Confocal microscopy images of H1838 cells incubated with HyApNc (A647N-**3** + A550-**4**) nanoconstructs (**a**; c2; red; A647N-**3** signal, **b**; c3: magenta; A550-**4** signal, **c**; c4; cyan; FRET signal measured, **d**; white; FRET signal calculated, **e**; overlay: c2 + c3+ c4 + cal. FRET + H33342 + Alexa488-WGA). **f**–**j** Cells incubated with HyApNc.mut (A647N.mut-**3** + A550-**4**) as a negative control (**f**; c2; red; A647N.mut-**3** signal, **g**; c3: magenta; A550-**4** signal, **h**; c4; cyan; FRET signal measured, **i**; white; FRET signal calculated, **j**; overlay: c2 + c3 + c4 + cal. FRET + H33342 + Alexa488-WGA). Both Atto647N (c2: Red) and Atto550 (c3: Magenta) fluorescence were observed from the cytosol including a FRET-mediated Atto647N signal, where the cells were incubated with HyApNc. In contrast, the mutilated functional nanoconstruct with Atto647N-labeled mutant trCLN3-L4 (A647N.mut-**3**) resulted in a very weak fluorescence signal for both dyes inside cells including a poor FRET signal. Reconstructed calculated FRET images for HyApNc (**d**) and HyApNc.mut (**i**) are given respectively. Scale bar: **a**–**j**, 50 µm

Time dependent UV exposure on cell mortality

In order to test the influence of time dependent UV exposure on cell mortality, H1838 cells were grown at different seeding densities of 10,000, 15,000, 20,000 and 30,000 cells per well in duplicates in a 96-well plate 24 h prior to the experiments. After 24 h of incubation at 37 °C in 5% CO_2-atmosphere, the cell medium was replaced with 100 µL of fresh RPMI medium. Each well containing a different cell density was exposed to UV irradiation of 365 nm for 0, 5, 10, 15 and 30 min, respectively, at a fixed intensity of 350 mW cm^{-2}. The cells were allowed to grow further for 24 h. Afterwards, 10 µL of an MTT stock solution (5 mg mL^{-1}) was added to each well, and the cells were incubated at 37 °C for 6 h. After labeling the cells with MTT, 100 µL of the SDS-HCL solution was added to each well, mixed thoroughly by use of a pipette, and incubated at 37 °C for an additional 12 h. Finally, the absorbance was measured at λ = 570 nm by using a Tecan Infinite® M1000 PRO microplate reader. The percentage of cell viability was determined by comparing the UV treated cells with the untreated control samples.

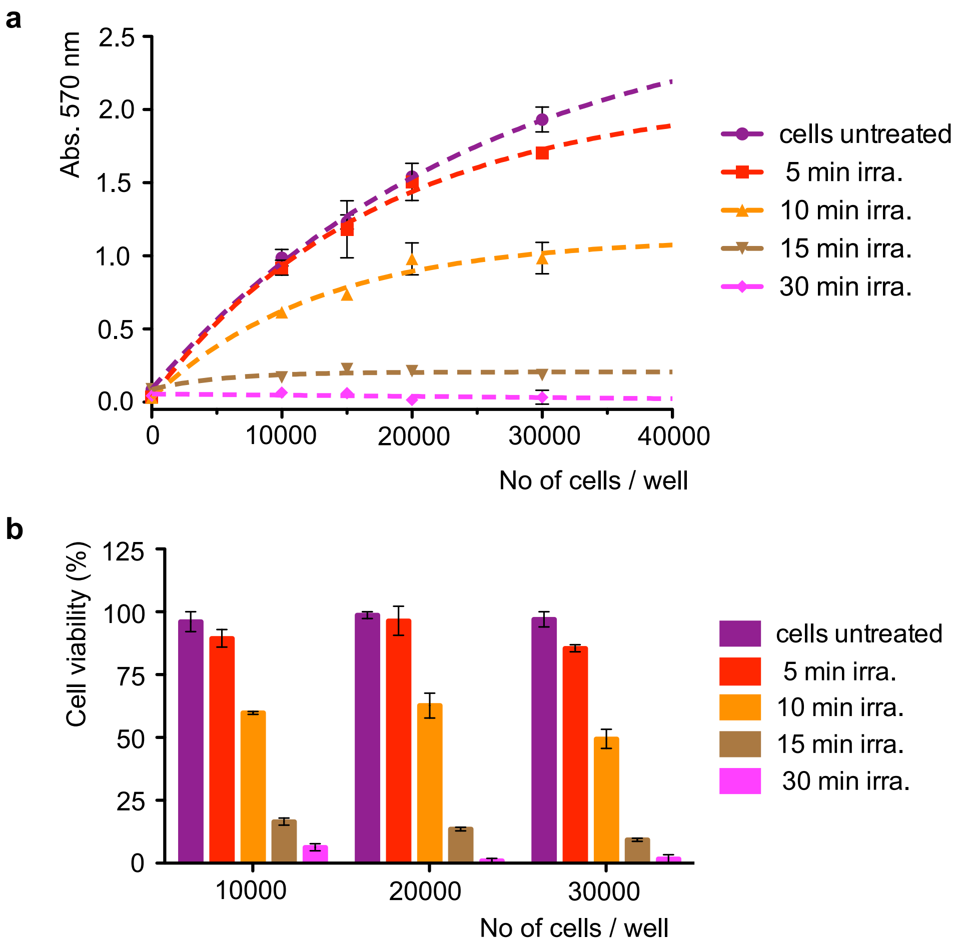

Supplementary Figure 20. Time dependent growth inhibition assay for H1838 cells exposed to UV light. **a** Cells irradiated at λ = 365 nm for 0 (●), 5 (■), 10 (▲), 15 (▼) and 30 (◆) min at a fixed intensity of 350 mW cm^{-2}. **b** Relative cell viability of H1838 cells at diferrent cell densities under different irradiation times (error bars: n = 2, mean ± S.D.)

Supplementary References

1. Kwak, M. et al. Virus-like particles templated by DNA micelles: a general method for loading virus nanocarriers. *J. Am. Chem. Soc.* **132**, 7834−7835 (2010).
2. Vinkenborg, J. L., Mayer, G. & Famulok, M. Aptamer-based affinity labeling of proteins. *Angew. Chem. Int. Ed.* **36**, 9176–9180 (2012).
3. Kikin, Oleg., D'Antonio, L. & Bagga, P. S. QGRS Mapper: a web-based server for predicting G-quadruplexes in nucleotide sequences. *Nucleic Acids Res.* **34**, W676−W682 (2006).
4. Uddin, G. & Azam, Z. A Novel Oligo-DNA Probe Carrying Non-nucleosidic Silylated Pyrene Derivatives : Synthesis and Excimer Forming Ability. *Am. J. Biochem. Mol. Biol.* **3**, 175−181 (2013).
5. Asanuma, H., Liang, X., Nishioka, H., Matsunaga, D., Liu, M. & Komiyama, M. Synthesis of azobenzene-tethered DNA for reversible photo-regulation of DNA functions: hybridization and transcription. *Nature Protocols* **2**, 203−212 (2007).
6. Stuart, C. H. et al. Site-Specific DNA–Doxorubicin Conjugates Display Enhanced Cytotoxicity to Breast Cancer Cells. *Bioconjugate Chem*. **25**, 406−413 (2014).
7. Kastantin, M. et al. Thermodynamic and Kinetic Stability of DSPE-PEG(2000) Micelles in the Presence of Bovine Serum Albumin. *J. Phys. Chem. B* **114**, 12632– 12640 (2010).
8. Dong, H. et al. 3-Helix micelles stabilized by polymer. *J. Am. Chem. Soc.* **134**, 11807–11814 (2012).
